# Relationship between klotho and physical function in healthy aging

**DOI:** 10.1038/s41598-023-47791-5

**Published:** 2023-11-30

**Authors:** Eliott Arroyo, Cecilia A. Leber, Heather N. Burney, Gayatri Narayanan, Ranjani Moorthi, Keith G. Avin, Stuart J. Warden, Sharon M. Moe, Kenneth Lim

**Affiliations:** 1 Division of Nephrology & Hypertension, Department of Medicine, Indiana University School of Medicine, Indianapolis, IN USA; 2 Department of Health and Exercise Science, Wake Forest University, Winston-Salem, NC USA; 3 Department of Chemistry and Biochemistry, University of Notre Dame, Notre Dame, IN USA; 4 Department of Biostatistics and Health Data Science, Indiana University School of Medicine, Indianapolis, IN USA; 5 Department of Physical Therapy, Indiana University School of Health and Human Sciences, Indianapolis, IN USA

**Keywords:** Biomarkers, Medical research, Pathogenesis

## Abstract

Epidemiological studies have reported a strong association between circulating Klotho and physical function; however, the cohorts were comprised of older adults with multiple comorbidities. Herein, we examined the relationship between Klotho and physical function in a community-based cohort of healthy adults. In this cross-sectional study, serum Klotho was measured in 80 adults who visited the Musculoskeletal Function, Imaging, and Tissue Resource Core of the Indiana Center for Musculoskeletal Health. Participants (n = 20, 10 [50%] men per group) were chosen into four age groups: 20–34, 35–49, 50–64, and ≥ 65 years, and were further grouped based on performance (low vs. high) on grip strength and chair stand tests. Klotho levels were lower in the ≥ 65 years group (703.0 [189.3] pg/mL; *p* = 0.022) and the 50–64 years group (722.6 [190.5] pg/mL; *p* = 0.045) compared to 20–34 years (916.1 [284.8] pg/mL). No differences were observed in Klotho between the low and high performers. The ≥ 65 years group walked a shorter distance during the 6-min walk test (6MWT) compared to 20–34 years (*p* = 0.005). Klotho was correlated with age (*p* < 0.001), body fat (*p* = 0.037), and 6MWT distance (*p* = 0.022). Klotho levels decline as early as the fifth decade of life, potentially before the onset of age-related impairment in exercise capacity.

## Introduction

The population of the United States of America is getting older. The nation’s 65 and older population is expected to nearly double over the next few decades, from 49 million in 2016 to 95 million people in 2060^1^. Moreover, more than 15% of people between the ages of 65–74 are reported to have ambulatory difficulty (serious difficulty walking or climbing stairs), along with 26% of those between the ages of 75–84, and 48% of those 85 and older^2^. The loss in physical function places a significant burden on affected individuals, their families, and the health-care system. Poor physical function is associated with lower quality of life^3^, loss of functional independence^4^, higher risk of hospitalization^5^, and increased mortality^6^. Furthermore, the total estimated cost of hospitalizations for US adults with self-reported functional limitations was a staggering $40 billion (value in year 2014)^7^. Beyond exercise and physical activity interventions, there are limited options for improving physical function. Therefore, identifying potential modifiable targets for preventing or reversing physical function decline is critically important. Accumulating data suggests that the “anti-aging” protein Klotho may play a key role in the development of functional impairments^8^.

α-Klotho, hereby referred to as Klotho, is a large ~ 135 kDa transmembrane glycoprotein that is predominantly expressed in the distal convoluted tubules of the kidneys^9^. Soluble forms of Klotho are released from the cell surface through ectodomain shedding by membrane proteases (ADAM10, ADAM17, and BACE1)^10,11^. Cleavage at the juxtamembrane site releases a 130 kDa soluble Klotho isoform containing both the KL1 and KL2 functional domains, whereas cleavage between the KL1 and KL2 domains releases a protein containing just the KL1 domain (70 kDa)^12^. A major function for Klotho is regulating mineral homeostasis by serving as an obligate co-receptor for fibroblast growth factor 23 (FGF23)^13^. A landmark study by Kuro-o et al.^14^, found that *Klotho*-deficient mice exhibited a shortened lifespan and a premature aging phenotype that included functional impairments, such as severe muscle wasting, hypokinesis, an abnormal walking pattern, and decreased stride length. In support of these findings, experimental models have shown that Klotho is involved in several key processes that regulate skeletal muscle function, such as muscle regeneration, mitochondrial biogenesis, oxidative stress, and inflammation^15–18^. Importantly, total circulating Klotho levels have been shown to decline with increasing age^19^, and several epidemiological studies in older adults—all of which included those with chronic diseases—have revealed a strong association between lower Klotho levels and increased disability in activities of daily living, increased risk of frailty, lower performance in the short physical performance battery (SPPB), lower grip strength, and lower knee strength^20–24^.

The majority of studies investigating the relationship between circulating Klotho and physical function focused solely on older adults and have included those with comorbidities^20–24^. The problem is that it is currently unclear whether circulating levels of Klotho are associated with physical function in individuals without comorbidities, and whether they are also associated with impairments in physical function earlier in life. The present study therefore sought to examine the relationship between serum Klotho levels and physical function indices in a community-based cohort of healthy adults across various age categories. Elucidating this relationship enables us to examine the natural history of age-related declines in circulating Klotho and its relationship with physical function in the absence of any chronic disease. We hypothesized that serum Klotho levels are associated with higher measures of physical function in all age groups.

## Methods

### Study design and cohort

In this cross-sectional study, we analyzed serum samples and data collected from 80 individuals who visited the Musculoskeletal Function, Imaging, and Tissue (MSK-FIT) Resource Core of the Indiana Center for Musculoskeletal Health (Indianapolis, IN, USA) between March 2018 and April 2019. Participants were recruited to the core by self-referral from the local community and by investigators seeking standardized physical function testing of their research participants.

To be eligible for inclusion in the present analyses, participants needed to be at least 20 years old, had provided a serum sample collected at the time of their MSK-FIT Core visit that was stored within the Indiana Biobank, had completed chair stand and dominant hand grip strength tests, and had no current diagnosis or prior history of hypertension, major metabolic disease, cardiovascular disease, pulmonary/respiratory disease, renal disease, or musculoskeletal disease.

Participants who had visited the MSK-FIT Core were stratified based on four stages of adult life: 20–34 (early adulthood), 35–49 (early middle age), 50–64 (late middle age), and ≥ 65 years (late adulthood)^25^. Individuals within each sex and age group were ranked for their performance on a hand grip strength test and a test of the number of chair stands completed in 30 s. The five individuals within each sex and age group with the lowest and highest composite rank were selected and grouped as low and high performers, respectively. Thus, in each of the four age groups there were equal numbers of females and males and equal numbers of low and high performers. This approach has been previously used to examine the association between β-aminoisobutyric acid (BAIBA) and physical performance^26^.

### Klotho

Total soluble Klotho levels were measured in serum using a well-validated solid-phase sandwich enzyme-linked immunosorbent assay (ELISA) (Cat. No. 27998, Immuno-Biological Laboratories, Inc., Minneapolis, MN, USA)^27^. To eliminate inter-assay variance, all samples for each assay were thawed once and analyzed in the same assay run by a single technician. All samples were analyzed in duplicate with an intra-assay coefficient of variation of 3.0%.

### Participant characteristics and body composition

Height (to nearest 0.1 cm) and weight (to nearest 0.1 kg) were measured without shoes using a calibrated stadiometer (Seca 264; Seca GmbH & Co., Hamburg, Germany) and scale (MS140-300; Brecknell, Fairmont, MN), respectively. Body fat mass (% and kg), lean mass (kg), appendicular lean mass relative to height squared (kg/m^2^), and bone mineral density (BMD; g/cm^2^) were assessed by whole-body dual-energy x-ray absorptiometry (DXA; Norland Elite; Norland at Swissray, Fort Atkinson, WI, USA). Regional DXA scans were performed to assess spine and hip BMD.

### Physical function

Participants completed a battery of physical function tests and questionnaires. Dominant hand grip strength (Jamar Plus + digital hand dynamometer; Sammons Preston, Bolingbrook, IL), number of chair stands completed in 30 s, and time to complete 5 chair stands were assessed, as we have previously described^28^. For grip strength testing, the participant sat upright in a chair with feet flat on the floor, elbow at 90 degrees of flexion, and forearm on the arm rest of the chair. The participant was asked to squeeze as hard as they could for a minimum of 3 s, keeping their trunk and arm stationary. The tester instructed the participant to “squeeze, squeeze, squeeze” with a consistent tone and volume. The peak reading was recorded, and the test repeated twice more with 30-s intervening rest periods. The maximum peak grip strength recorded from the 3 trials was used in analyses. Time to walk 4-m from a stationary start at normal speed (usual gait speed) was measured with a stopwatch and converted to speed (m/s), as we have previously reported^29^. Static balance was assessed by testing the ability to balance for 10 s with feet in side-by-side, semi-tandem, and tandem positions. For each stance, the tester first demonstrated the feet position. They then supported the participant by the arm while the participant positioned their feet as was demonstrated. After the participant confirmed they were ready for the test to begin, their arm was released, and the stopwatch was started. The test was terminated once the subject reached for support, took a step, or 10 s elapsed. Each position was tested once and the duration for each position was recorded. The results from the tests of time to complete 5 chair stands, usual gait speed, and static balance were used to calculate the SPPB score out of 12^30^. A higher SPPB score indicates better function.

For the 6-min walk test (6MWT), participants were instructed to walk around two cones separated by 20 m along a corridor, whereby one lap (from cone A to cone B and back) was 40 m^31^. Participants were instructed to walk quickly and complete as many laps as possible in 6 min. The total distance covered during the test was recorded.

### Self-reported function and physical activity levels

Self-reported physical function was assessed using physical function (PF) domain of the National Institutes of Health Patient-Reported Outcomes Measurement Information System computerized adaptive test (PROMIS PF CAT; version 1.2) and the PF domain of the Short Form-36 (SF-36 PF) questionnaire.

Self-reported physical activity (PA) levels were obtained by the 7-day short-form International Physical Activity Questionnaire (IPAQ)^32^. The questionnaire collects information on time spent walking, in moderate-intensity PA, in vigorous-intensity PA, and sitting (sedentary time). Responses were used to estimate total weekly PA per week (MET·min/wk), calculated as duration × frequency per week × metabolic equivalent of task (MET) intensity (walking = 3.3 METs; moderate-intensity PA = 4.0 METs; vigorous-intensity PA = 8.0 METs). The total physical activity MET-minutes per week and total hours of sedentary time per week were reported.

### Study endpoints

Our primary endpoint was physical function status (low versus high performers). The secondary endpoints were scores in the PROMIS PF CAT, physical performance (gait speed, chair stand test performance, 6MWT distance, grip strength), and body composition (body fat % and appendicular lean mass).

### Statistical analysis

Descriptive statistics were employed to summarize participant characteristics and measures. Continuous variables were summarized by mean (standard deviation [SD]) if normally distributed, or median (interquartile range [IQR]) otherwise. Categorical variables were summarized by frequency (relative frequency in percentage). Age group comparisons were analyzed using one-way analysis of variance (ANOVA) followed by Tukey’s post hoc test for pairwise comparisons or by Kruskal–Wallis test followed by Dunn’s post hoc test, as appropriate. Comparisons between low and high performers in each age group were analyzed using independent samples *t*-test or Mann–Whitney U test, as appropriate. Pearson’s correlation coefficients were calculated to identify factors associated with Klotho levels. p-values < 0.05 were considered statistically significant and missing observations were excluded. All analyses were performed using R Statistical Software (v4.1.1, Vienna, Austria).

### Ethics statement

The Musculoskeletal Function, Imaging, and Tissue (MSK-FIT) Resource Core of the Indiana Center for Musculoskeletal Health (Indianapolis, IN, USA) has Institutional Review Board approval (IRB study #170755085) from Indiana University for sample and data collection and storage, and additional Indiana University Institutional Review Board approval was obtained for the current analyses. All participants provided written informed consent before participating in the MSK-FIT Core allowing their deidentified samples and data to be stored and retrieved. All methods were performed in accordance with the relevant guidelines and regulations.

## Results

### Study population characteristics

Characteristics of the study population are shown in Table 1. Both the 65 + years group (*p* = 0.022) and the 50–64 years group (*p* = 0.045) had lower circulating Klotho levels compared to the 20–34 years group. The 65 + years group had higher fat mass compared to the 20–34 years group (*p* = 0.028) and lower total BMD compared to the 20–34 years group (*p* = 0.013) and the 35–49 years group (*p* = 0.008). Total hip BMD T-score was also lower in the 65 + years group compared to both the 20–34 years group (*p* < 0.001) and the 35–49 years group (*p* = 0.003), and it was also lower in the 50–64 years group compared to the 20–34 years group (*p* = 0.005). Similarly, femoral neck BMD T-score was lower in the 65 + years group compared to both the 20–34 years group (*p* < 0.001) and the 35–49 years group (*p* < 0.001), and it was also lower in the 50–64 years group compared to the 20–34 years group (*p* < 0.001).There were no age group differences in BMI, body fat %, lean mass, or appendicular lean mass (all *p* ≥ 0.05).

**Table 1:** Participant characteristics.

Characteristics	20–34 years n = 20	35–49 years n = 20	50–64 years n = 20	65 + years n = 20	*P*-value^a^
Male, n (%)	10 (50)	10 (50)	10 (50)	10 (50)	1.00
Age, years	25.0 (22.0–29.2)	43.0 (38.0–47.0)*^‡^	55.0 (52.0–60.5)*^†^	70.5 (65.8–75.5)*^†‡^	< 0.001
Height, cm	172.0 (10.1)	172.9 (9.1)	170.7 (9.1)	167.8 (7.2)	0.30
Weight, kg	72.6 (10.4)	82.2 (12.1)	79.3 (11.6)	78.5 (16.6)	0.13
BMI, kg/m^2^	24.4 (22.3–25.6)	26.7 (24.2–30.8)	26.8 (24.8–29.7)	26.5 (24.2–29.4)	0.06
Body Fat, %	25.9 (11.9)	29.0 (11.0)	32.6 (11.2)	34.5 (10.0)	0.08
Fat Mass, kg	14.1 (11.0–21.9)	20.4 (14.2–26.0)	19.4 (17.4–29.0)	23.2 (19.4–28.4)*	0.035
Lean Mass, kg	43.4 (39.0–59.1)	50.0 (44.7–57.4)	47.2 (37.5–52.3)	47.9 (36.8–53.0)	0.40
Appendicular Lean Mass, kg/m^2^	7.8 (1.5)	8.2 (0.9)	7.8 (1.6)	7.7 (1.3)	0.72
Total BMD, g/cm^2^	1.03 (0.14)	1.05 (0.12)	0.97 (0.11)	0.91 (0.12)*^†^	0.004
Spine (L1-L4) BMD T-Score	− 0.14 (1.12)	0.17 (1.18)	− 0.18 (0.96)	− 0.43 (1.26)	0.48
Total Hip BMD T-Score	0.37 (1.14)	0.00 (1.25)	− 0.92 (1.23)*	− 1.41 (1.04)*^†^	< 0.001
Femoral Neck BMD T-Score	0.19 (1.56)	− 0.51 (1.04)	− 1.23 (1.25)*	− 2.01 (0.95)*^†^	< 0.001
Klotho, pg/mL	916.1 (284.8)	829.6 (239.2)	722.6 (190.5)*	703.0 (189.3)*	0.015

Data shown as mean (standard deviation) if normally distributed, or median (interquartile range) otherwise.

BMI, body mass index; BMD, bone mineral density.

^a^*p* value for overall group effect.

*Significant difference from 20 to 34 years age group.

^†^Significant difference from 35 to 49 years age group.

^‡^Significant difference from 50 to 64 years age group.

Participants in each age group were further stratified into high and low performers (Table S1). The high performers in the 20–34 years group had a higher total BMD compared to the low performers (*p* = 0.023). Additionally, the high performers in both the 50–64 years group (*p* = 0.005) and the 65 + years group (*p* = 0.029) were younger when compared to the low performers in their respective groups. The high performers in the 50–64 years group had higher appendicular lean mass when compared to the low performers (*p* = 0.022).

### Physical function

Physical function measures in each age group are shown in Table 2. The 65 + years group walked a shorter distance in the 6MWT compared to the 20–34 years group (*p* = 0.005) and reported lower physical function on the SF-36 PF compared to the 20–34 years group (*p* = 0.001) and the 35–49 years group (*p* = 0.027). The 65+ years group also scored lower on the PROMIS PF CAT compared to the 20–34 years group (*p* = 0.004) and the 35–49 years group (*p* = 0.024). The 65+ years group reported lower sedentary time compared to the 20–34 years group (*p* = 0.007). There were no age group differences in grip strength, chair stand performance, usual gait speed, SPPB scores, or PA levels (*p’s* ≥ 0.05).

**Table 2:** Measures of physical function.

	20–34 years	35–49 years	50–64 years	65+ years	p-value^a^
Grip strength, kg	36.7 (32.6–44.3)	39.5 (31.4–45.6)	35.4 (26.7–40.3)	28.5 (20.8–36.7)	0.10
Time to complete 5 chair stands, s	8.2 (6.1–11.1)	7.8 (5.8–9.8)	7.8 (6.9–10.9)	8.8 (6.8–13.0)	0.30
Chair stands completed in 30 s, n	18.5 (12.8–24.3)	19.0 (14.3–24.5)	17.0 (12.0–22.0)	16.0 (10.8–21.0)	0.18
Usual gait speed, m/s	1.4 (1.3–1.5)	1.4 (1.2–1.6)	1.4 (1.3–1.5)	1.3 (1.2–1.4)	0.16
Distance walked in 6 min, m	638.5 (560.0–680.5)	612.0 (552.0–653.8)	596.0 (547.5–619.0)	496.0 (435.5–587.5)*	0.007
SPPB score	12 (12–12)	12 (12–12)	12 (12–12)	12 (10–12)	0.14
SF-36 PF score	100 (98.8–100)	100 (93.8–100)	100 (90–100)	92.5 (75–96.3)*^†^	0.002
PROMIS PF CAT T Score	60.0 (7.3)	58.5 (8.6)	57.7 (7.5)	51.7 (6.0)*^†^	0.004
Total PA, MET·min/wk	4878.0 (3069.0–7518.0)	5238.0 (4580.0–8269.1)	3357.8 (2467.1–7138.5)	4123.5 (2536.5–6167.6)	0.23
Sedentary time, h/wk	8.0 (6.5–8.0)	6.0 (4.8–8.0)	6.0 (4.8–8.0)	5.0 (3.8–6.3)*	0.014

Data shown as mean (standard deviation) if normally distributed, or median (interquartile range) otherwise.

SPPB, short physical performance battery; SF-36 PF; the physical function domain of the Short Form-36 questionnaire; PROMIS PF CAT, the physical function domain of the National Institutes of Health Patient-Reported Outcomes Measurement Information System computerized adaptive test; PA, physical activity.

^a^*p* value for overall group effect.

*Significant difference from 20 to 34 years age group.

^†^Significant difference from 35 to 49 years age group.

As expected, when stratified by performance category (Table S2), the high performers in each age group had greater grip strength, better performance on the chair stand tests, and higher scores in the PROMIS PF CAT when compared to the low performers in their respective age groups (all *p* < 0.05). The high performers in the 20–34 years, 50–64 years, and 65 + years age groups also exhibited faster usual gait speed and greater distance walked in the 6MWT compared to the low performers in their respective age groups (all *p* < 0.05); however, no significant differences in usual gait speed (*p* = 0.85) or distance walked in the 6MWT (*p* = 0.05) were observed between the high performers and low performers in the 35–49 years group. There were no differences in PA levels or sedentary time between high and low performers in any age group (all *p* ≥ 0.05).

### Relationship between serum klotho and age, body composition, and physical function

**Figure 1:**
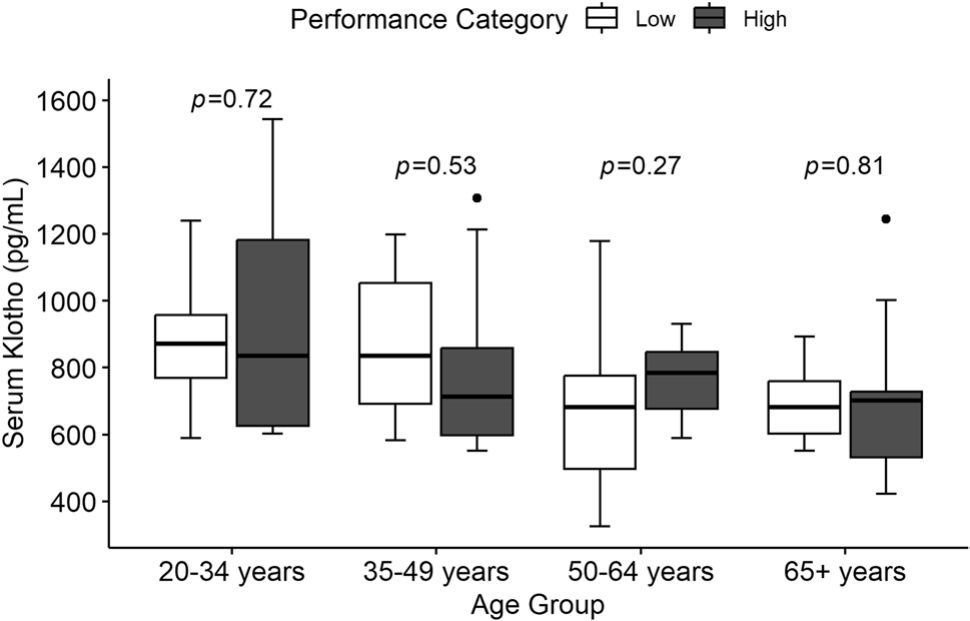
Differences in serum Klotho between low and high performers. *P* values are displayed for comparison between performance category in each age group. No differences were observed in serum Klotho levels between the high performers and low performers in any age group.

Serum Klotho levels in high and low performers in each age group are shown in Fig. 1 and Table S1. No differences in circulating Klotho levels were observed between the low and high performers in any age group.

**Figure 2:**
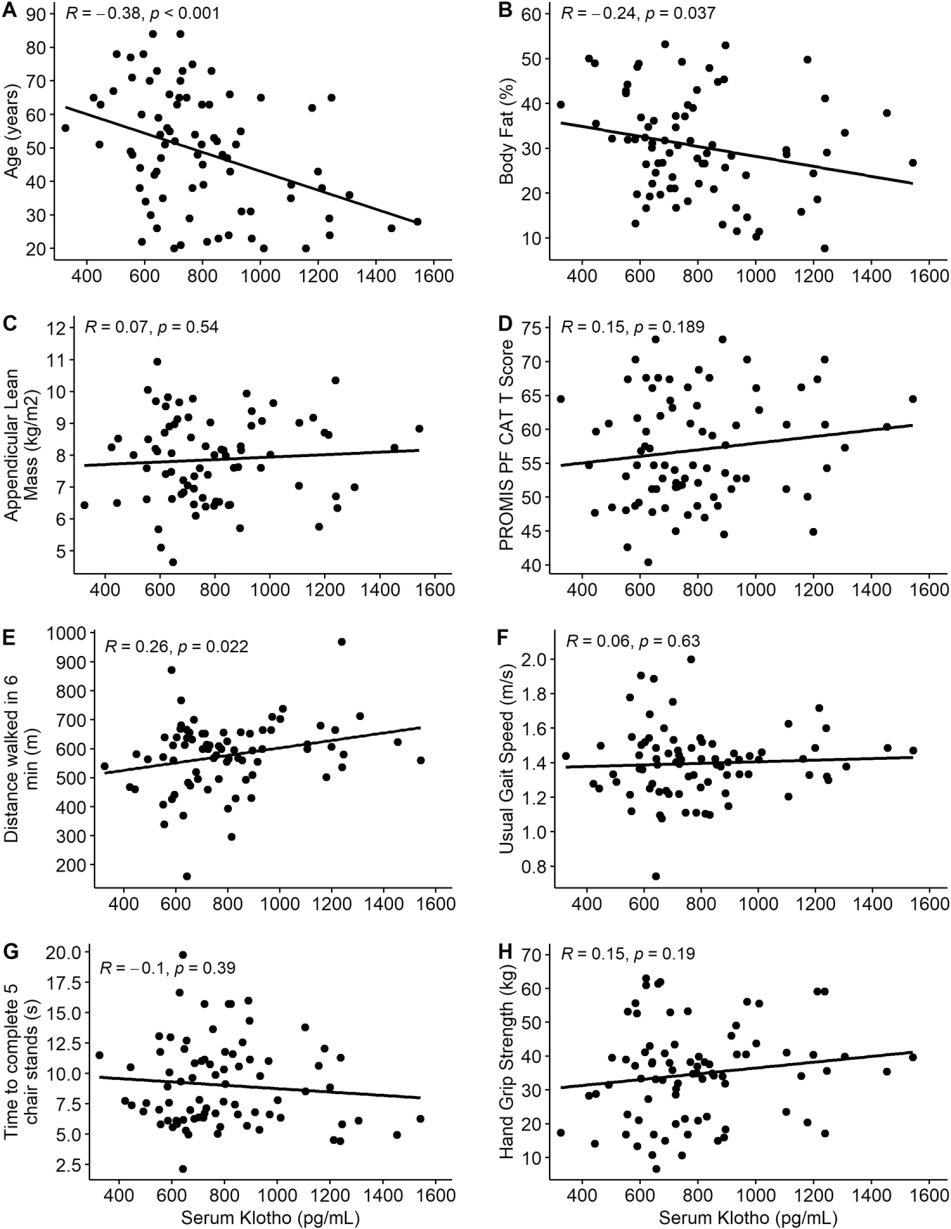
Associations between serum Klotho and age, body composition, and physical function indices. (**A**) Age. (**B**) Body fat %. (**C**) Appendicular lean mass. (**D**) Score on the physical function domain of the National Institutes of Health Patient-Reported Outcomes Measurement Information System computerized adaptive test (PROMIS PF CAT). (**E**) Distance walked during 6-min walk test. (**F**) Usual gait speed. (**G**) Time to complete 5 chair stands. (**H**) Grip strength. Serum Klotho was significantly associated with age, body fat %, and distance walked in the 6-min walk test.

Correlation analyses were employed to assess the relationship between Klotho levels and our secondary endpoints (Fig. 2). Serum Klotho was associated with age (*r* = − 0.38, *p* < 0.001), body fat % (*r* = − 0.24, *p* = 0.037), and distance walked in the 6MWT (*r* = 0.26, *p* = 0.022). Serum Klotho was not correlated with PROMIS PF CAT score (*r* = 0.15, *p* = 0.19), usual gait speed (*r* = 0.06, *p* = 0.63), time to complete 5 chair stands (*r* = − 0.10, *p* = 0.39), grip strength (*r* = 0.15, *p* = 0.19), or appendicular lean mass (*r* = 0.07, *p* = 0.54).

## Discussion

The present study examined the relationship between serum Klotho levels and physical function in a cohort of healthy adults across various age groups. Our findings indicate that serum Klotho levels decline with increasing age as early as the fifth decade of life even in the absence of chronic health conditions. Moreover, only the 65 years and older age group exhibited significantly lower performance in the 6MWT and lower self-reported PROMIS and SF-36 physical function compared to the 20–34 years group, which suggests that declines in circulating Klotho may precede declines in physical function with aging.

Data from the Invecchiare in Chianti (Aging in the Chianti Area; InCHIANTI) study, a population-based longitudinal study of older adults (median age = 75 years) in Italy, showed that plasma Klotho levels were independently associated with grip strength in participants with plasma Klotho < 681 pg/mL^20^. This suggests that 681 pg/mL may represent a critical threshold for Klotho deficiency below which muscle strength begins to decline in older adults. However, 30 out of 80 participants (37.5%) in the present study had Klotho levels < 681 pg/mL, of which 16 were classified as “high performers” in their age groups based on their grip strength and repeat chair stand test performance. Therefore, whether a critical threshold for Klotho deficiency exists remains unclear. Moreover, another study in the InCHIANTI study cohort found that plasma Klotho levels were significantly associated with SPPB score^24^. In contrast, we found no significant differences in serum Klotho levels between the high and low performers in any age group. This is likely attributed to the differences in age and functional status between the two cohorts. Even the 65 + age group in the present study had a lower median age (70.5 years) compared to the InCHIANTI study cohort (75.0 years) and only 4 participants in the present study had an SPPB score < 10, whereas the mean (SD) SPPB score in the InCHIANTI study was 8.2 (2.4) and 7.6 (3.0) in participants with plasma Klotho > 669 pg/mL and ≤ 669 pg/mL, respectively^24^. An SPPB score < 10 is predictive of all-cause mortality^33^ and a score ≤ 8 has been established as the cutoff for severe sarcopenia^34^. Only one participant in our cohort met the criteria for confirmed sarcopenia based on the latest recommendations from the European Working Group on Sarcopenia in Older People^34^, and none of the participants met the criteria for “severe” sarcopenia. Hence, the relationship between Klotho levels and physical function may be limited to those with clinically significant physical function impairment.

Serum Klotho levels were inversely correlated with age and body fat %, which is consistent with previous studies that have found that circulating Klotho levels decline with increasing age^19,27^. Moreover, a randomized controlled trial investigating the effects of different training modalities on plasma Klotho levels in sedentary middle-aged adults living in Spain found that exercise training-induced increases in circulating Klotho were significantly associated with decreases in body fat %^35^. This suggests that increases in Klotho may underlie improvements in body composition following exercise training. To this end, it has recently been suggested that Klotho may be a novel “exerkine,” a soluble factor released in response to exercise training that contributes to the health benefits associated with physical activity^8,36^. In support of this hypothesis, experimental studies in obese^37^ and aged^38^ mice have found that Klotho treatment leads to significant reductions in adiposity and improvements in muscle strength. This may be due to the role of Klotho in regulating skeletal muscle mitochondrial function and oxidative metabolism^16^. It has also been suggested that exercise training-induced increases in circulating Klotho are mediated by peroxisome proliferator-activated receptor-γ (PPAR-γ), a ligand-activated nuclear transcription factor essential for glucose and lipid metabolism^39^, which is also upregulated in response to exercise training^40^. It should be noted that we found no significant differences in self-reported PA levels between the high performers and low performers in any age group, which may partly explain the similar circulating Klotho levels between the high performers and low performers.

We found that Klotho levels were positively correlated with performance in the 6MWT. Although cardiopulmonary exercise testing (CPET) is considered the gold standard for assessing functional capacity, the 6MWT is a simple field test shown to have strong prognostic value in patients with cardiovascular disease^41,42^. Consistent with our findings, data from the InCHIANTI study revealed that higher plasma Klotho levels are associated with a lower risk of cardiovascular disease^43^. Moreover, we previously showed that Klotho knockdown in vascular smooth muscle cells (VSMC) promoted the development of accelerated VSMC calcification, a common feature of cardiovascular disease in aging and chronic kidney disease (CKD)^44^. To this end, circulating Klotho has been considered as a biomarker of cardiovascular disease in patients with CKD, which is a known state of Klotho deficiency^45^. In the present study, both the 65 + years group and the 50–64 years group had significantly lower circulating Klotho levels compared to the 20–34 years group, though only the 65+ years group exhibited significantly lower performance in the 6MWT compared to the 20–34 years group. Therefore, declines in circulating Klotho may precede the onset of cardiovascular disease with aging, which further supports the use of Klotho as a potential biomarker of cardiovascular disease.

A strength of the present study is that it provides a comprehensive assessment of physical function and body composition that includes both subjective and objective measures of physical function as well as DXA derived measures of lean and fat mass, and BMD. Another strength is that the low and high performers were at the extreme ends of physical performance within their age group, thereby potentiating the identification of physiological differences. Moreover, this is the first study to-date to examine the relationship between Klotho and physical function in a healthy cohort of adults across various stages of life. A limitation of the study is the relatively small sample size in each age category. The high variability in Klotho levels in humans may require a larger sample size to detect statistically significant differences in Klotho levels between the low and high performers. Future prospective studies with larger sample sizes are needed to further evaluate the relationship between circulating Klotho and changes in physical function across lifespan. Another limitation of our study is the use of convenience sampling, which can introduce selection bias and limit the generalizability of our findings. Problems with commercially available Klotho assays may also contribute to the discrepancies in our findings^46^. Studies have found that the type of sample, method of collection and storage, and number of freeze–thaw cycles can all influence assay performance and yield a wide range of results^47–49^. Although a immunoprecipitation–immunoblot (IP–IB) assay has recently been shown to have better overall performance when compared to the ELISA used herein^47^, the labor-intensive nature of the IP–IB assay limits its utility. Furthermore, the present study did not control for exercise, dietary intake, time of day, nor menstrual cycle when collecting blood samples, which are factors that are known to influence circulating concentrations of Klotho and may therefore also contribute to the high variability in serum Klotho concentrations reported herein.

## Conclusions

The present study examined the relationship between circulating Klotho and indices of physical function and body composition in healthy adults ranging from 20 to 34 years old to 65 years and older. The findings indicate that Klotho levels decline as early as the fifth decade of life but cannot distinguish between healthy adults with high and low physical function. We postulate that the relationship between Klotho levels and physical function may be limited to certain conditions, such as those with clinically significant physical function impairment.

### Supplementary Information

Supplementary Tables.

## Data Availability

Data for the results of this study are available from the corresponding authors upon reasonable request.
