# Supplementary material for: Relationship between klotho and physical function in healthy aging
Source: Sci Rep. 2023 Nov 30;13:21158. doi: 10.1038/s41598-023-47791-5 (PMC10689840; doi:10.1038/s41598-023-47791-5)
Supplement: Supplementary file 1 — Supplementary Tables. [file 41598_2023_47791_MOESM1_ESM.docx]

# SUPPLEMENTAL MATERIAL

Table S1. Participant Characteristics Stratified by Performance Category

Table S2. Measures of Physical Function Stratified by Performance Category

Table S1. Participant Characteristics Stratified by Performance Category

|  | **20-34 years** | | **35-49 years** | | **50-64 years** | | **65+ years** | |
| --- | --- | --- | --- | --- | --- | --- | --- | --- |
|  | **Low** | **High** | **Low** | **High** | **Low** | **High** | **Low** | **High** |
|  | **n = 10** | **n = 10** | **n = 10** | **n = 10** | **n = 10** | **n = 10** | **n = 10** | **n = 10** |
| **Male, n (%)** | 5 (50) | 5 (50) | 5 (50) | 5 (50) | 5 (50) | 5 (50) | 5 (50) | 5 (50) |
| **Age, y** | 23.5  (22.0-27.8) | 27.0  (23.8-29.8) | 42.5  (39.0-44.5) | 43.5  (38.0-47.8) | 60.5  (56.0-63.0) | 52.5*  (51.3-54.0) | 74.0  (71.5-77.8) | 66.5*  (65.0-70.0) |
| **Height, cm** | 169.5 (10.2) | 174.6  (9.8) | 172.9 (11.0) | 172.8  (7.3) | 168.0  (6.8) | 173.4 (10.7) | 166.1  (7.3) | 169.6  (6.9) |
| **Weight, kg** | 69.2  (9.3) | 75.9  (10.9) | 84.8  (11.7) | 79.7  (12.5) | 78.4  (8.8) | 80.2  (14.2) | 80.2  (16.1) | 76.8  (17.8) |
| **BMI, kg/m^2^** | 23.7  (22.6-24.8) | 24.8  (22.5-26.7) | 28.7  (24.6-31.8) | 25.2  (24.0-30.5) | 27.1  (24.7-30.4) | 26.1  (25.1-29.3) | 27.5  (25.5-31.7) | 25.4  (22.3-28.7) |
| **Body Fat, %** | 28.8  (13.2) | 23  (10.3) | 31.8  (11.6) | 26.7  (10.6) | 36.8  (10.9) | 28.4  (10.4) | 38.2  (8.1) | 30.3  (10.7) |
| **Fat Mass, kg** | 14.1  (11.6-22.6) | 15.3  (10.7-20.9) | 22.7  (17-28.7) | 20.4  (13.4-24.9) | 23.5  (18.1-33.2) | 18.7  (17-21.7) | 24.2  (22.6-35.5) | 20.3  (15.8-26.1) |
| **Lean Mass, kg** | 39.8  (37.5-48.6) | 50.7  (42.9-59.7) | 48.8  (42.3-58.8) | 50  (45.6-55.7) | 38.3  (34.6-50.3) | 50.1  (42-60.7) | 45.9  (35.7-52) | 47.9  (37.1-53.8) |
| **Appendicular Lean Mass, kg/m^2^** | 7.4  (1.2) | 8.2  (1.6) | 8.0  (0.9) | 8.3  (0.9) | 7.0  (1.3) | 8.6*  (1.5) | 7.8  (1.4) | 7.7  (1.2) |
| **Total BMD, g/cm^2^** | 0.965 (0.139) | 1.102* (0.102) | 1.024 (0.117) | 1.066 (0.124) | 0.939 (0.116) | 1.009 (0.084) | 0.891 (0.114) | 0.939 (0.136) |
| **Spine BMD (L1-L4) T-Score** | -0.6  (0.9) | 0.3  (1.2) | 0.2  (1.4) | 0.1  (1.1) | -0.3  (0.8) | -0.0  (1.1) | -0.2  (1.3) | -0.7  (1.3) |
| **Total Hip BMD T-Score** | -0.0  (1.1) | 0.7  (1.1) | -0.3  (1.7) | 0.2  (0.9) | -1.1  (1.2) | -0.7  (1.3) | -1.6  (1.1) | -1.2  (0.9) |
| **Femoral Neck BMD T-Score** | -0.3  (1.4) | 0.7  (1.6) | -0.8  (1.4) | -0.3  (0.8) | -1.6  (1.2) | -0.9  (1.3) | -2.2  (1.1) | -1.8  (0.7) |
| **Klotho, pg/mL** | 892.2 (195.5) | 939.9 (363.0) | 864.6 (214.4) | 794.6 (268.5) | 673.5 (240.7) | 771.7 (115.4) | 692.5 (117.2) | 713.4 (248.3) |

Data shown as mean (standard deviation [SD]) if normally distributed, or median (interquartile range [IQR]) otherwise.

Low = low performers. High = high performers. Abbreviations: BMI, body mass index; BMD, bone mineral density.
*Significant difference from Low performers in the respective age group

Table S2. Measures of Physical Function Stratified by Performance Category

|  | **20-34 years** | | **35-49 years** | | **50-64 years** | | **65+ years** | |
| --- | --- | --- | --- | --- | --- | --- | --- | --- |
|  | **Low** | **High** | **Low** | **High** | **Low** | **High** | **Low** | **High** |
|  | **n = 10** | **n = 10** | **n = 10** | **n = 10** | **n = 10** | **n = 10** | **n = 10** | **n = 10** |
| **Grip Strength, kg** | 31.8  (18.2-34.2) | 47.7*  (38.1-58.4) | 30.4  (19-40.3) | 46.6*  (38.1-55.1) | 24.6  (17.0-35.2) | 42.2*  (35.6-51.7) | 20.5  (16.8-22.6) | 37.7*  (31.6-41.1) |
| **Time to complete 5 chair stands, s** | 11.1  (10.6-13.1) | 6.0*  (5.6-6.3) | 10.2  (9-12.4) | 5.8*  (5.1-6.1) | 10.9  (10.4-11.9) | 6.9*  (5.7-7.6) | 13 .0  (11.6-15.4) | 6.8*  (6.3-7.5) |
| **Chair stands completed in 30 s, n** | 12.5  (10.5-14.0) | 24.5*  (23.0-26.5) | 13.5  (11.3-15.0) | 25.0*  (24.0-29.0) | 12.0  (11.3-14.5) | 22.0*  (20.3-24.0) | 10.5  (9.3-11.8) | 21.0*  (18.5-22.0) |
| **Usual Gait Speed, m/s** | 1.3  (1.3-1.4) | 1.5*  (1.4-1.6) | 1.4  (1.2-1.5) | 1.4  (1.3-1.7) | 1.3  (1.3-1.4) | 1.5*  (1.4-1.5) | 1.2  (1.1-1.3) | 1.4*  (1.3-1.5) |
| **Distance walked in 6 min, m** | 569.0  (542.0-648.8) | 667.0*  (637.8-731.3) | 583.0 (498.0-612.3) | 646.5 (615.5-662.8) | 540.0  (502.0-596.0) | 600.0*  (575.8-645.8) | 435.5 (379.5-487.0) | 581.0*  (496.0-615.0) |
| **SPPB Score** | 12  (11-12) | 12*  (12-12) | 12  (11-12) | 12*  (12-12) | 12  (11.3-12) | 12  (12-12) | 10  (9-11) | 12*  (12-12) |
| **SF-36 PF Score** | 97.5  (95-100) | 100*  (100-100) | 95  (91.3-100) | 100  (100-100) | 90  (86.3-100) | 100  (96.3-100) | 77.5  (55-88.8) | 95*  (95-100) |
| **PROMIS PF CAT T Score** | 55.3  (6.5) | 64.6*  (4.6) | 54.1  (7.0) | 62.9*  (7.9) | 53.7  (6.6) | 61.7*  (6.2) | 48.0  (4.5) | 55.4*  (5.1) |
| **Total PA, MET·min/wk** | 3192.0 (2868.0-7998.0) | 6119.0 (4495.5-6898.3) | 5878.5 (4938.0-8152.13) | 4799.0 (4516.0-8327.3) | 3037.5 (1935.8-5692.8) | 4199.3 (2815.5-7231.5) | 4201.5 (2122.5-5652.8) | 3990.0 (3181.5-7958.7) |
| **Sedentary time, h/wk** | 8.0  (7.0-11.0) | 7.0  (6.3-8.0) | 6.0  (4.3-7.8) | 6.5  (5.0-7.8) | 6.5  (5.3-8.0) | 6.0  (3.5-7.5) | 5.0  (4.0-5.8) | 4.5  (3.0-6.8) |

Data shown as mean (standard deviation [SD]) if normally distributed, or median (interquartile range [IQR]) otherwise.

Low = low performers. High = high performers. Abbreviations: SPPB, short physical performance battery; SF-36 PF; the physical function domain of the Short Form-36 questionnaire; PROMIS PF CAT, the physical function domain of the National Institutes of Health Patient-Reported Outcomes Measurement Information System computerized adaptive test; PA, physical activity.
*Significant difference from Low performers in the respective age group
